# The FIRE-IN project: Tsunami-risk related practitioner challenges and 3rd cycle overall results

**DOI:** 10.12688/openreseurope.15249.1

**Published:** 2023-01-10

**Authors:** Georgios Sakkas, Ioannis Tsaloukidis, Danai Kazantzidou-Firtinidou, Iris Schneider, Vassiliki Kouskouna, Nico Hybbeneth, Claudia Berchtold, Juliane Schlierkamp, Marta Miralles, Sebastien Lahaye, Michel Bour

**Affiliations:** 1 Center for Security Studies, Sector of Emergency Management and Civil Protection, 4 Kanellopoulou str., Athens GR-10177, Greece; 2 German Federal Agency for Technical Relief (THW) Headquarters, Unit E I 3, Research Projects, Provinzialstraße 93 D 53127 Bonn, Germany; 3 Section of Geophysics-Geothermics, National and Kapodistrian University of Athens, Panepistimiopolis – Zographou, 17584 Athens, Greece; 4 Department for Technology Analysis and Strategic Planning (TASP), Unit for Public Technology and Innovation Planning (TIP), Fraunhofer Institute for Technological Trend Analysis – INT, Appelsgarten 2, 53879 Euskirchen, Germany; 5 Catalan Department of Interior, Catalan Fire and Rescue Service, Carrer de la Diputació, 355, 08009, Barcelona, Spain; 6 SAFE Cluster, Domaine du Petit Arbois, Avenue Louis Philibert - BP 10028, 13545 Aix en Provence Cedex, France

**Keywords:** FIRE-IN, tsunami, Mediterranean, Europe, future challenges, capacity building, resilient societies, practitioners

## Abstract

This article summarizes the methodology for the identification of practitioners’ challenges of the H2020 funded project FIRE-IN (Fire and Rescue Innovation Network) activities with a strong focus on the natural hazard mitigation working group and tsunamis in the Mediterranean region as a case study for the 3rd cycle. The scenario of a tsunami occurrence in the Mediterranean is the basis for the FIRE-IN 3rd cycle workshop, as an indicative example of a high impact – low probability event, which aims to identify the Future Common Capability Challenges of practitioners in Europe. The current status of the tsunami hazard in Europe, national and international tsunami risk mitigation measures and procedures and operational experience from recent events are also discussed. Focus is provided on the natural hazard mitigation and tsunami related practitioners’ challenges, while results from the FIRE-IN request for ideas process and the interaction between practitioners, researchers and industry is also discussed. The aim is to present the current and future capability challenges of practitioners, one of the main outcomes of FIRE-IN project, and to provide further guidelines to stakeholders of disaster management towards a safer Europe, mainly, through preparedness for stronger and resilient societies.

## Disclaimer

The views expressed in this article are those of the authors. Publication in Open Research Europe does not imply endorsement of the European Commission.

## Introduction

FIRE-IN, the Fire and Rescue Innovation Network, is a Horizon 2020 project that establishes and supports a network of practitioners, technological providers and researchers with a main goal to improve the national and European Fire and Rescue capability development process and consequently enhance the security level of the citizens of the European Union. The objectives of FIRE-IN are to create a strong network of practitioners, researchers, industry and standardization organizations and to provide a Strategic Research Agenda for Europe, based on capability-driven approaches through the practitioners’ operational needs. Through its outcomes, the project aims at supporting the European Commission policy makers to build the Research and Innovation Agenda. The main activities within the project are (a) the identification and harmonization of operational capability gaps, (b) the scouting of promising solutions, (c) the request for ideas by the community and the various relevant stakeholders and (d) the definition of a Fire and Rescue Strategic Research and Standardization Agenda. The work of FIRE-IN is clustered in five Thematic Working Groups (TWGs): (A) Search and Rescue Emergency Response, (B) Structure Fires, (C) Landscape Fire Crisis Mitigation, (D) Natural Hazard Mitigation and (E) Chemical Biological Radiological Nuclear Explosives (CBRNE).

The work is organized in three cycles over a timeframe of five years. The first two cycles of the project were devoted to the study of the current Common Capability Challenges (CCCs) with a horizon timeframe of 5–10 years ( Gallardo *et al*., 2019; Lahaye *et al*., 2018), while the third cycle is devoted to the study of Future Common Capability Challenges (FCCCs) with a horizon timeframe for the next 10–15 years ( Miralles *et al*., 2021). This paper focuses on the 3rd cycle, the identification of the FCCCs for the Natural Hazard Mitigation TWG ( Miralles *et al*., 2021). First a brief overview of the project is presented, introducing the methodology of FIRE-IN project for identifying practitioners’ capability challenges, the screening and request for ideas and the challenges of the first cycle. Tsunamis as a risk for Europe are presented along with a justification for their selection as a case study for Europe during the 3 ^rd^ cycle of the project, the status for mitigation and preparedness actions in Europe and worldwide related to tsunamis as well as the recent operational experience due to earthquake induced tsunamis in Greece and the Aegean Sea. Tsunamis are examined as a major potential threat (high impact – low probability events) for Europe for the next 10 to 15 years, inside the context of the Natural Hazard Mitigation Working Group, with the aim to identify significant future capability challenges practitioners are facing. A scenario of mega-earthquake in the Mediterranean with a cascading tsunami that can affect a significant part of Europe is the basis for the discussion, review and elaboration of the results of the 3rd cycle of workshops for the Natural Hazard Mitigation TWG (D) as already defined in respective project deliverables ( Miralles *et al*., 2021). Current tsunamigenic zones in Europe, national and international tsunami risk mitigation measures, recent operational experience based on international literature as well as the identified challenges are presented in the following sections. The identified future common capability challenges for the natural hazards working group are presented along with an overall discussion on the results of the project for the 3 ^rd^ cycle with a focus to natural hazards based on already published deliverables of the project ( Tsaloukidis *et al.*, 2022). Overall, the aim is to present the new capability challenges for practitioners, to provide their current status in terms of coverage via technologies, standards and research items, and to discuss the significance of turn towards preparedness against response, through the fundamental change of culture of practitioners, decision-makers as well as the citizens for a safer and more resilient Europe.

## FIRE-IN methodology for the identification of practitioners’ challenges, screening of solutions and request for ideas

The identification of CCCs and FCCCs is based on a methodology that was developed jointly for all TWGs in order to ensure proper taxonomy, workflow and coherence between the five TWGs. In each cycle, the five TWGs work along those lines by organizing, implementing and evaluating the results of dedicated workshops per TWG with experts from the aforementioned areas (practitioners, researchers and industry).

### The general framework

The approach for the methodological framework is based on the ACRIMAS taxonomy and framework project ( Stolk *et al*., 2012). Crisis management functions are clustered in “preparatory”, “operational” and “common” tasks and it is used to define capability challenges and end-users needs. The mapping of operational, supporting and preparatory tasks during crisis management that can cover all crisis management phases is the first step for the identification of practitioners’ challenges. Each TWG pinpoints the main challenges, the key interest areas, interesting scenarios and potential networks. The methodology and stakeholders’ engagement are described in deliverable D1.1 ( Linde-Frech *et al*., 2018).

Operational tasks include topics such as on-site crisis mitigation, search and rescue, law enforcement, evacuation and sheltering, information and involvement of the general public, health services, supply and restoration of basic infrastructure ( Linde-Frech *et al*., 2018). Supporting tasks include command and control, coordination, decision making and planning, situational awareness, information management, monitoring and logistics ( Linde-Frech *et al*., 2018). Preparatory tasks include policy making, capacity and capability building, training and exercises, procurement procedures, maintenance, warehousing, raise of the community awareness, lessons learnt and evaluation ( Linde-Frech *et al*., 2018). Operational and supporting tasks refer to the response and recovery phases, while preparatory tasks to mitigation and preparedness.

### Identification of CCCs through workshops

Each working group follows the implementation of the tasks suggested in the general framework. This process was carried out through a workshop implementation and a common reporting template ( Gallardo *et al*., 2019; Lahaye *et al*., 2018; Miralles *et al*., 2021) that examines the background of the “problem”, the opportunity for improvements, i.e., the capability challenges, any potential constraints or already identified best practices, the opportunities from various perspectives such as, procedures, personnel, equipment, tools and technologies. All TWGs implement the procedure for the identification of CCCs based on the discussion of specific scenarios and the aforementioned tasks.

### Screening and request for ideas

After the identification of the FIRE-IN Common Capability Challenges, a screening process initiates for each capability and challenge, in the research, standardisation and technology domain in order to seek existing solutions that address the specific challenges. Further to that, a “Request for ideas” process initiates to identify the level of coverage of each challenge and capability by existing solutions. This process is supported by a schema that was developed through the project, the traffic light system ( Salvi & Frecenon, 2017) with which every challenge is examined against technology, research items and standards and certain criteria, in order to examine how well covered the challenge can be considered. The “Request for Ideas” is a process in which relevant to crisis to research and development stakeholders can provide information and solutions that can address specific CCCs or FCCCs, either in the form of an existing solution or in the form of a future product. The latter step is crucial as it provides a good overview whether a challenge can be covered by existing solutions and thus provide input for new uncovered areas.

### Common Capability Challenges

By the process mentioned above, it has been found that the five TWGs share common problems and that the three types of tasks (preparatory, operational and common) for the crisis management functions of practitioners could be similar. Thus, the identified challenges that practitioners face are clustered in four dimensions:

a) the “High Flow of effort in hostile environment”,
b) the “Low frequency, high impact events”,
c) the “Multi-agency / multi-leadership environment” and
d) the “High level of uncertainty”.

In addition, the capabilities identified are clustered in seven topics, which are:

a) the “Incident Command Organization”,
b) “Pre-planning”,
c) “Guidance instruments”,
d) “Knowledge cycle”,
e) “Information management”,
f) “Community involvement” and
g) “Technology”.

**Figure 1.**
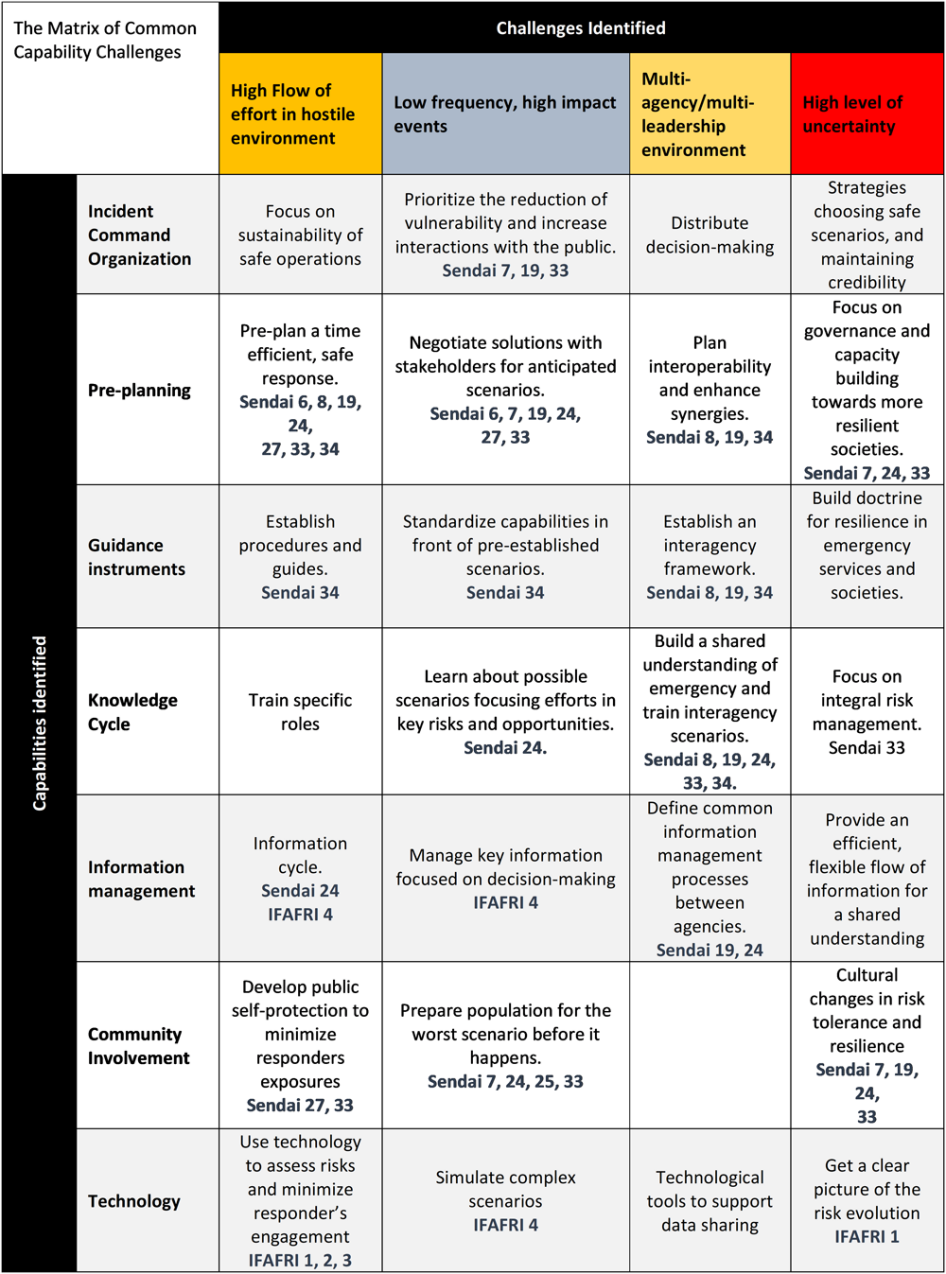
The Common Capability Challenges matrix of the FIRE-IN project as published during the 1st cycle of the project ( Lahaye *et al*., 2018). Sendai X refers to the respective article, out of the 50, presented in the Sendai Framework. IFAFRI X refers to the respective IFAFRI gap.

The challenges and the capabilities are considered within the various tasks and from the Common Capability Challenges matrix, which is common for the five TWGs. In the first cycle of the project, 27 Common Capability Challenges that are depicted in Figure 1 ( Lahaye *et al*., 2018) were identified by the practitioners. More information about the 27 CCCs can be found in the FIRE-IN e-platform. These challenges are in accordance with the International Forum to Advance First Responder Innovation (IFAFRI) gaps and the Sendai Framework targets and priorities to prevent new disaster risks and reduce existing ones ( Sendai, 2015).

During the 2nd cycle of the project, it was decided to focus on the detailed explanation of the first cycle's 27 CCCs and their prioritization from the practitioner side of view. This led to a list of twelve CCCs in the areas of Community Involvement, Technology, Knowledge Cycle and Incident Command Organization, which were considered as the most important ( Gallardo *et al*., 2019).

## TWG-Natural Hazards Mitigation: Tsunamis as a case study for Europe

The thematic working group of natural hazard mitigation of the FIRE-IN project focuses on the study of natural hazards and on the identification of challenges in this topic. The first cycle of the project focused on flash floods, the second cycle focused on winter storm events and the third cycle, which is the main discussion item of this study, focused on tsunamis.

According to the recent study of the European Commission document regarding the natural and man-made disaster risk for Europe ( EC DG-ECHO, 2021), in which the national risk assessments of member states are considered, the top five disaster risks of concern to national authorities and at EU level are extreme weather events, floods, wildfires and geophysical risks. Other risks that receive considerable attention in national reports, as well as in the report of the Commission, include epidemics, disruption of critical infrastructure, terrorism, cyber threats and animal and plant diseases.

FIRE-IN natural hazard mitigation working group selected tsunamis as the main topic of discussion within its 3rd cycle of activities, acknowledging that tsunamis have been recognized as an imminent risk for Europe for the next 10–15 years and in addition to the following facts:

a) The scenario must be based on a cross-border hazard that can affect multiple nations.
b) Cascading events should be considered to the maximum possible extent.
c) The focus of the scenario should target to the future, not depicting the most usual situation, thus being a low probability – high impact one, but at the same time being realistic based on past events.
d) Tsunami risk is considered in the national risk assessment studies of 2021 ( EC DG ECHO, 2021) of almost all countries of southern Europe, compared to the equivalent studies of 2017 ( EC DG ECHO, 2017). For example, Italy and Spain did not consider tsunamis as a potential risk in 2015 but they included in the 2018/2019 study.
e) The devastating events of Crete-Greece (365), Rhodes-Greece (1303) and Messina – Italy (1908) are not so frequent, but the overall risk is high. In addition, the extreme events of the last twenty years in Sumatra (2004), Chile (2010) and Japan (2011) as well as the recent event of Samos-Greece (2020) are perfect reminders of the imminent risk and thus, mitigation and preparedness are the best tools to deal with it.
f) The losses, human and economic, that can be inflicted by tsunamis, can be considerably high, and the 2011 Japan earthquake and tsunami is the most representative example for this, as the measurable consequences in impacts cost is more than 200 billion US$, and the affected lives are approximately 19,000 people ( Kouskouna *et al*., 2021).

A Tsunami scenario fits perfectly with the above-mentioned criteria to identify the Future Common Capability Challenges (FCCCs) and, since not a major event has been recorded for many years in Europe, it is something of interest for the future. The recent event in Samos ( Triantafyllou *et al*., 2021) proved that the selection of the examination of a tsunami event in Mediterranean has a rationale as areas of Europe may be significantly affected.

### Tsunami sources in Europe

Tsunamis are frequent in Europe and occur mainly in the South, in the Mediterranean Sea, and in the West, in the Atlantic Ocean (e.g., Alvarez-Gomez *et al.*, 2011; Ambraseys & Synolakis, 2010; Basili *et al*., 2021). Thus, the most vulnerable countries to tsunamis are Greece, Portugal, Spain, Italy and France. According to Sørensen *et al*., (2012), the tsunami related hazard in the Mediterranean region is usually estimated considering the “most credible” scenarios for limited geographical regions, with little attention being paid to the probability of occurrence of any given scenario which may lead to underestimation of tsunami hazard in the area, while tsunamis of high inundation heights have been observed. In terms of hazard, and according to Sørensen *et al*. (2012), Eastern Mediterranean presents a higher hazard compared to the Western part, both in terms of probability of occurrence of tsunamis and in terms of tsunami heights. More specifically, tsunami heights can reach up to 20m, in Southwestern Greece, for return periods of 2000 years and up to 8m for moderate return periods of 500 years. Similarly, the probability of a tsunami occurrence of 1m in 30 years is higher than 50% in Southwestern Greece ( Sørensen *et al*., 2012).

Active tectonics dominate the Eastern Mediterranean and many earthquake-induced tsunamis have been recorded and observed in the area. Various scientific studies, such as Ambraseys (1962); Ebeling *et al*. (2012); Galanopoulos (1960); Papadopoulos & Chalkis (1984); Papadopoulos & Fokaefs (2005); Papadopoulos *et al*. (2007); Papazachos & Papazachou (2003); Petricca & Babeyko (2019); Tinti *et al*. (2001); Triantafyllou *et al*. (2021), examine tsunamis in the Mediterranean, in Portugal and in Europe overall. Ambraseys & Synolakis (2010) note the fact that many studies related to tsunamis require a careful approach for historic events, especially before 1928. Papadopoulos *et al*. (2007) identify 18 tsunami events of high intensity in the Eastern Mediterranean for the period from 1948 up to 2002, with a likely mean recurrence interval of 142 years. Papazachos & Papazachou (2003) in their study identify 83 tsunami events from 479 B.C. up to 1999 A.D. related to earthquakes. Tinti *et al*. (2001) have created an updated catalogue of tsunami events for Europe consisting of 94 reliable identified events.

Two well-known tsunamis that affected the greater part of the Mediterranean are the events of 365 A.D. in the Southwest of Crete, and of 1303 A.D. east of the Rhodes Island, both caused by earthquakes with magnitude M≥8 (e.g., Tinti *et al*., 2005). Another characteristic example of large tsunamis is the one caused by the 1956 earthquake of Amorgos with magnitude M=7.5 and inundation height up to 25m in the southern coast of Amorgos island, 20m in the northern coast of Astypalaia and 2.6m in the northern coast of Crete island ( Papazachos & Papazachou, 2003). An example of tsunami not caused by an earthquake but from a landslide which was triggered by heavy rainfalls, is the 7 February 1963 event, in the Gulf of Corinth ( Papazachos & Papazachou, 2003). The case of the 1600 BC eruption of Thera volcano caused a remarkably strong tsunami that destroyed the Minoan civilization ( Friedrich *et al*., 2006). Also, the 1650 eruption of a submarine volcano off the coast of Santorini caused a tsunami ( Papazachos & Papazachou, 2003).

Baptista & Miranda (2009) present a revision of the catalogue of tsunamis in Portugal. From 60 BC to 1980, 18 events have been recorded. The most studied tsunami in Portugal is the one triggered by the 1755 Great Lisbon earthquake with its epicenter 200 km away from Lisbon in the Atlantic Οcean ( Zitellini *et al*., 1999). Southern Spain is also at high risk for tsunamis according to the studies of Birkman *et al*. (2010) and Alvarez-Gomez *et al*. (2011). Tinti *et al*. (2005) have simulated specific scenarios of earthquakes with triggered tsunamis in South Italy, Greece and Algeria.

Tsunami memories have been awakened by the earthquake of 2017 of Kos ( Heidazardeh *et al*., 2017), when a tsunami hit the island of Kos with a maximum runup of 1.5m (in Bodrum-Asia Minor the maximum was 1.9m), the recent events of 2020 in Samos due to the Mw=7.0 earthquake, which hit the coast of Samos with a maximum runup of 2.8m at Ayios Nikolaos, 2m at Vathy (capital of Samos) ( Triantafyllou *et al*., 2021) and Asia Minor with a maximum runup of 3.8 at Akarca ( Dogan *et al*., 2021), as well as, the Mw=6.6 earthquake south of Crete, that generated a small tsunami with a rise and drop of the sea around 25 cm in 2020 in Ierapetra and 35 cm in Chrysi islet ( Papadopoulos *et al*., 2020).

These events although located in the Eastern Mediterranean can affect the entire Mediterranean Sea. For example, Amato (2020) clearly shows how the first tsunami wave, created by the 24 October 2018 Mw=6.7 earthquake in Zakynthos Greece ( Papadimitriou *et al*., 2021), can reach the coast of Gibraltar in 270 minutes without any significant alert. It is characteristic to observe the colour-coded alert levels issued by the Italian National Centre for Geophysics and Volcanology (INGV) that Amato (2020) presents. A red alert level was issued for the Ionian islands (Zakynthos, Kefallinia, Lefkada) in Greece, yellow for various other locations of Greece, as well as for locations in Albania and South Italy, while for the rest sites in the Mediterranean a green alert level was issued. According to Tinti *et al*. (2005) and based on specific scenarios, an earthquake in the Mediterranean and especially in North Algeria of magnitude Mw=6.9, could hit the Balearic islands in 30 minutes and in 45 to 60 minutes the coast of South Eastern Spain practically affecting the Western Mediterranean. According to the same study, an earthquake of magnitude Mw=7.4 offshore Sicily can affect Southern parts of Italy in 30 to 45 minutes, while an earthquake in the Eastern Hellenic Arc can affect almost the entire Eastern Mediterranean Sea.

However, tsunamis also occur as a result of volcanic eruptions and submarine landslides ( Paris *et al.*, 2020). About five percent of the total number of tsunamis recorded since 1600 can be attributed to volcanoes ( Paris *et al*., 2014).

At present, it must be stated that no operational early warning system in the world can take landslides into account. While for tsunamis triggered by earthquakes, special software based on earthquake monitoring and satellite data can warn of a tsunami within minutes. There are also active volcanoes near the sea in Europe. The danger of a tsunami in the event of an eruption or the slipping of a flank should therefore not be underestimated here either.

### Current status of EU and international tsunami risk mitigation actions

Tsunamis are a cascading natural phenomenon that are recorded in many places around the globe, especially in active tectonic areas. Large tsunami events, such as the ones of 2004 in the Indian Ocean (e.g., Rabinovich *et al*., 2006), 2009 in Samoa (e.g., Lay *et al*., 2010), 2010 in Chile (e.g., Martínez *et al*., 2017) and 2011 in Tohoku Japan (e.g., Koshimura *et al*., 2014), are the most characteristic examples of their devastating impacts to human civilization. Experience forced humanity to take on measures in order to reduce the impacts and increase the resilience of societies in case of tsunami occurrence. These measures are clustered in five major categories of measure on local or regional scale: (a) land-use planning, (b) building codes, (c) early warning systems along with evacuation planning, (d) structural defense measures and (e) risk awareness and assessment.

The Intergovernmental Oceanographic Commission of UNESCO (IOC-UNESCO) coordinates a global initiative, IOC-UNESCO Tsunami Programme, with the main goal to protect human lives from tsunamis. The programme supports all member states in the form of assessing tsunami risk, installing and implementing early warning systems for tsunamis, and providing the necessary education and training to communities at tsunami risk, focusing on preparedness measures. IOC-UNESCO is supported by (a) the various tsunami service providers, covering all the sea surface of the Earth, from the Pacific Ocean, the Caribbean Sea, Indian Ocean and North-Eastern Atlantic up to the Mediterranean Sea, which monitor seismic and sea level activity, and (b) the tsunami information centers which provide technical and capacity building assistance, education and outreach to member states and the public in preventing, preparing, and mitigating measures for tsunamis.

In Europe, the IOC of North-Eastern Atlantic, the Mediterranean and connected seas (NEAMTWS), along with the support of the national tsunami warning centers, monitors tsunami activities in the areas surrounding Europe and proposes mitigation and preparedness measures, through the establishment of an initial tsunami warning system and specific and common action plans in the case of a tsunami occurrence.

Another important initiative is the TsunamiReady Program established in the United States in 2001 by the US National Weather Service (NWS) of the National Ocean and Atmospheric Administration (NOAA), with the main goal to encourage and recognize tsunami preparedness of the community and minimize the risk posed by tsunamis in communities, through improved risk assessment, planning, education and warning communications. This initiative has been expanded at an international level through the collaboration of the NWS and the IOC-UNESCO. The TsunamiReady Recognition programme publishes specific guidelines in three languages, English, Spanish and French.

The Joint Research Centre of the European Commission initiated the “Tsunami Last Mile” project ( EC-JRC, 2019), with funding from the European Commission department for civil protection and humanitarian aid (DG-ECHO), after the two 2017 (June 12th and July 20th) tsunami events in the Aegean Sea, which includes specific infrastructure for tsunamis, such as sensors and stations that monitor seismic waves and sea level changes and public information boards, with the aim to create an early warning system for tsunamis in the Mediterranean Sea and stimulate public awareness for tsunamis. The main goal of the project was tsunami messages to definitely reach the general public.

Currently, Europe is being monitored, in terms of tsunamis and relevant early warning messages, by four national warning centers: (a) The CENtre d’Alerte aux Tsunamis (CENALT) in France, (b) the Portuguese Sea and Atmosphere Institute (IPMA) in Portugal, (c) the Italian National Centre for Geophysics and Volcanology (INGV) and (d) the Hellenic National Tsunami Warning Center of the National Observatory of Athens. In addition, tsunami warnings are also issued by the Turkish Kandili Observatory and Earthquake Research Institute of Istanbul (KOERI) which supports the monitoring in the Eastern Mediterranean Sea area.

At this point, it must be noted that the majority of early warning systems for tsunamis focus their efforts on warning the population in case of an earthquake triggered tsunami. Most existing systems work in parallel with networks of seismographs and accelerographs, thus excluding the cases of tsunamis induced by landslides or volcanic eruptions.

The TSUNAMI RISK project (full title: TsunamiRisk - Multi-risk assessment and cascade effect analysis in cooperation between Indonesia and Germany - Joint research on volcano- and landslide-induced tsunamis) funded by the German Federal Ministry of Education and Research addresses exactly this issue. The overall goal is to improve the Indonesian early warning system. Such an early warning system has been successfully integrated into the warning and decision-making chains since 2011. Nevertheless, a small percentage of tsunamis caused by these cascading events cannot be detected by the existing early warning system. Such a case is a large flank collapse of a volcano on Anak Krakatau in 2018 that led to a landslide and meter-high tsunami waves that hit coastal areas of the islands of Java and Sumatra largely unprepared and caused a high number of casualties ( Grilli *et al.*, 2019). The project attempts to close this capability gap. Results can subsequently be transferred to other areas of the world. Despite the fact that global and European initiatives have emerged, and, furthermore, historical events and scientists warn about future tsunami events, tsunami risk awareness, especially in Europe, is poor. This has also been pointed out by Karagiannis & Synolakis (2015) but it must be admitted that, in recent years, the efforts on tsunami risk awareness are more intense, coherent and targeted to the general public.

One of the most promising tools for risk awareness is the implementation of exercises in order to train both emergency management services and the general public on how to prepare for tsunamis, and how to act during a tsunami event. Although, exercises that target emergency services are implemented regularly, e.g., in the framework of research projects ( Karagiannis & Synolakis, 2015), with interesting results, however, exercises concerning the education of the public are still limited. The “Tsunami Last Mile” project organized a local exercise in the island of Kos, Greece, in order to enhance tsunami risk awareness to the public ( EC-JRC, 2019). On the other hand, raising public awareness with communication campaigns and exercises for the risk of earthquakes is considerably more advanced. For example, in Greece, the earthquake risk perception is significantly higher in the general public compared to the tsunami risk perception (e.g., the survey in EEFIT, 2021), proving that an exercise is a valuable tool for embedding risk awareness.

In building design codes, such as Eurocode 1 ( EN 199-1-6, 2005) and ASCE 7-16 (2016), water loading is considered among the actions to design for. More precisely, water effects caused by an earthquake (tsunamis) are hydrodynamic variable actions and are applied perpendicularly to the contact surfaces, taking into account the speed, water depth and shape of the structure. Bearing elements of large surface, which are perpendicular to the horizontal water loading are the most vulnerable to tsunami effects as they suffer from out-of-plane dynamic impact, being in principle the infill walls of reinforced concrete structures, masonry walls with low mortar quality or timber panels, where present. However, although water loading is described among actions on structures, no specific provisions for designing for building resistance are available at national or European level. As discussed also by Karagianis & Synolakis (2015), seismic design codes cannot be directly adopted for protection against water impact, due to the different load distribution, dynamic effects and most importantly the nature of the loads impact on the structure. As a result, there is lack of tsunami (or other dynamic water actions) resistant provisions for new or existing structures, what will be of primary prevention measure for urban protection against tsunamis.

Urban planning, control of the land use and building restrictions in coastal prone areas are some of the disaster risk mitigation measures, that although often regulated, have difficulties in their implementation, due to the existing urban fabric and the financial interest coastal areas are often associated with. On the other hand, countries, frequently suffering by tsunamis, e.g., Japan, have invested in other tangible mitigation measures along the coastlines, such as breakwaters and seawalls. However, due to the construction and maintenance cost, these are less popular mitigation measures for lower income states. Nevertheless, existing coastal structures designed to withstand sea waves to a certain limit, have not proved sufficient for larger tsunami waves, for which special structures are needed ( Karagiannis & Synolakis, 2015).

Risk assessment has been generally recognized as one of the tools offering scientific support to the operational disaster management planning and, if appropriately designed and informed, it may provide invaluable insights to be used both at prevention and at response phases. Tsunamis, especially in the Mediterranean, are considered a hazard of low probability - high impact and scientific research is expanded, further, to monitoring systems and hazard assessment, to vulnerability and risk assessment of exposed communities, despite the involved uncertainties. The latter require probabilistic or scenario-based hazard analysis, to be combined with structural and/or societal vulnerability to tsunami actions (with special fragility curves), yielding probable expected damage and associated losses. Although equivalent approaches are well advanced for seismic risk, a number of gaps is still recognized for tsunami risk studying ( Behrens *et al*., 2021), associated with physical as well as social vulnerability estimations, creation of comprehensive exposure and consequence datasets and identification of risk metrics. Hence, risk studies mostly remain at scientific level, without being yet adopted for disaster planning and preparedness at local or national level.

### Recent operational experience from Greece

The two tsunami events recorded in 2020 in Greece, triggered by the Mw=6.6 earthquake, on the 2nd May 2020, south of Ierapetra, Crete, and by the Mw=7.0 earthquake, on the 30th of October 2020 in Samos, respectively, revealed some interesting gaps in the central mechanism and its design. Inundation height was of the order of a few centimeters for the first case, while for the second case it reached 3.35 meters ( Triantafyllou *et al*., 2021).

These two events were an important test for the installed and operational tsunami early warning system operated from the Hellenic National Tsunami Warning Centre (HL-NTWC) as well as for the entire civil protection mechanism in Greece. Three different institutes monitor tsunami activity in the Eastern Mediterranean Sea. All three of them operate according to the specifications set out by NEAMTWS in the framework of IOC-UNESCO and provide their alerts to the national authorities and the European Emergency Response Coordination Centre (ERCC), but not directly to the general public. In Greece, alerts to the general public, for imminent and potentially disastrous natural hazards events, are issued only by the General Secretariat for Civil Protection. The monitoring activity and the alert issuing is based on (a) earthquake instrumental records and (b) a decision matrix with certain criteria according to the earthquake magnitude and epicentral distance, as no deep-ocean tide gauges are installed in the Mediterranean Sea ( Papadopoulos *et al*., 2020). The absence of deep ocean tide gauges is considered to be a major instrumentation gap for Europe, both at a scientific and operational level. All three monitoring institutes issue the same type of alert messages; the “Initial” message, the “Ongoing”, the “End” message and the “Cancel” message.

### Ierapetra earthquake induced tsunami, May 2020

Papadopoulos *et al*. (2020) in their study discuss both the efficiencies and deficiencies of the tsunami early warning system for the Ierapetra, Crete island (Greece), earthquake of May 2020. Their findings are very interesting as they point out significant gaps in preparedness and response and are useful for the identification of FCCCs.

One of the deficiencies of the early warning system was the timing of alert issuing. Three institutes, the Geodynamic Institute of the National Observatory of Athens, Greece (GEIN-NOA), the Institute of Geophysics and Volcanology in Italy (INGV) and the KOERI, recorded the earthquake and issued an alert with a difference of 1 to 4 minutes. This time difference is extremely crucial in cases the coast is close to the epicenter. The initiation of the tsunami was observed as a sea retreat at Ierapetra approximately five minutes after the alert issued by NOA, while the peak amplitude was recorded fourteen minutes after the initial message ( Papadopoulos *et al.*, 2020).

Another important deficiency, of the entire response mechanism, was the delay of the Greek authorities to issue the initial alert message for a couple of hours. At the same time, Papadopoulos *et al*. (2020) point out that despite the delay, a step forward has been made as it was the first time that such a message has been issued. In Israel, where the epicentral distance is larger compared to Greek sites, the respective tsunami travel time was more than one hour for the city of Haifa, but the response of the authorities was somehow delayed due to their deciding to proceed to the next message type, stay on an “advisory” message type or “cancel” the event. Finally, the event was cancelled at least 23 minutes before the arrival, nevertheless, this time is not adequate enough to evacuate on time to higher grounds largely populated areas.

Another important topic that is mentioned by Papadopoulos *et al*. (2020) is the absence of real-time scientific simulations that could support the decision making in minimizing the alert time. Real-time simulation can offer valuable information regarding the wave arrival time and inundation heights, thus enhancing the preparedness and guiding the response of emergency services by pointing out areas in risk.

Finally, one of the most interesting topics is related to society and population, as it was found that the risk awareness in the general public, regarding tsunamis, is not adequate. For example, from various videos and photos across the media, it was easily seen that people moved to the beach to observe the sea retreat and oscillations, acting in exactly the opposite way compared to the one provided in international guidelines in the tsunami cases, for self-protection measures.

### Samos earthquake induced tsunami, October 2020

This case is the most recent tsunami triggered by a damaging earthquake in the Eastern Mediterranean Sea. The northern coasts of Samos island were hit by two waves with a varying time difference from the earthquake occurrence depending on the epicentral distance. The difference in the nearest location (approximately 12km) was only 2 minutes, while in Samos capital (approximately 20km) was 19 minutes ( Triantafyllou *et al*., 2021). In this case also, the distance between the coast to the tsunami generation area is small, thus making early warning extremely important. It must be also noted that the tsunamis hit the coast of Smyrna (Izmir) across Samos island. According to the daily reports of ERCC daily map ( EU, 2020), the tsunami wave was also recorded in the islands of Lesvos to the North, the island of Syros to the West and the island of Kos to the South, approximately one hour after the earthquake.

**Figure 2.**
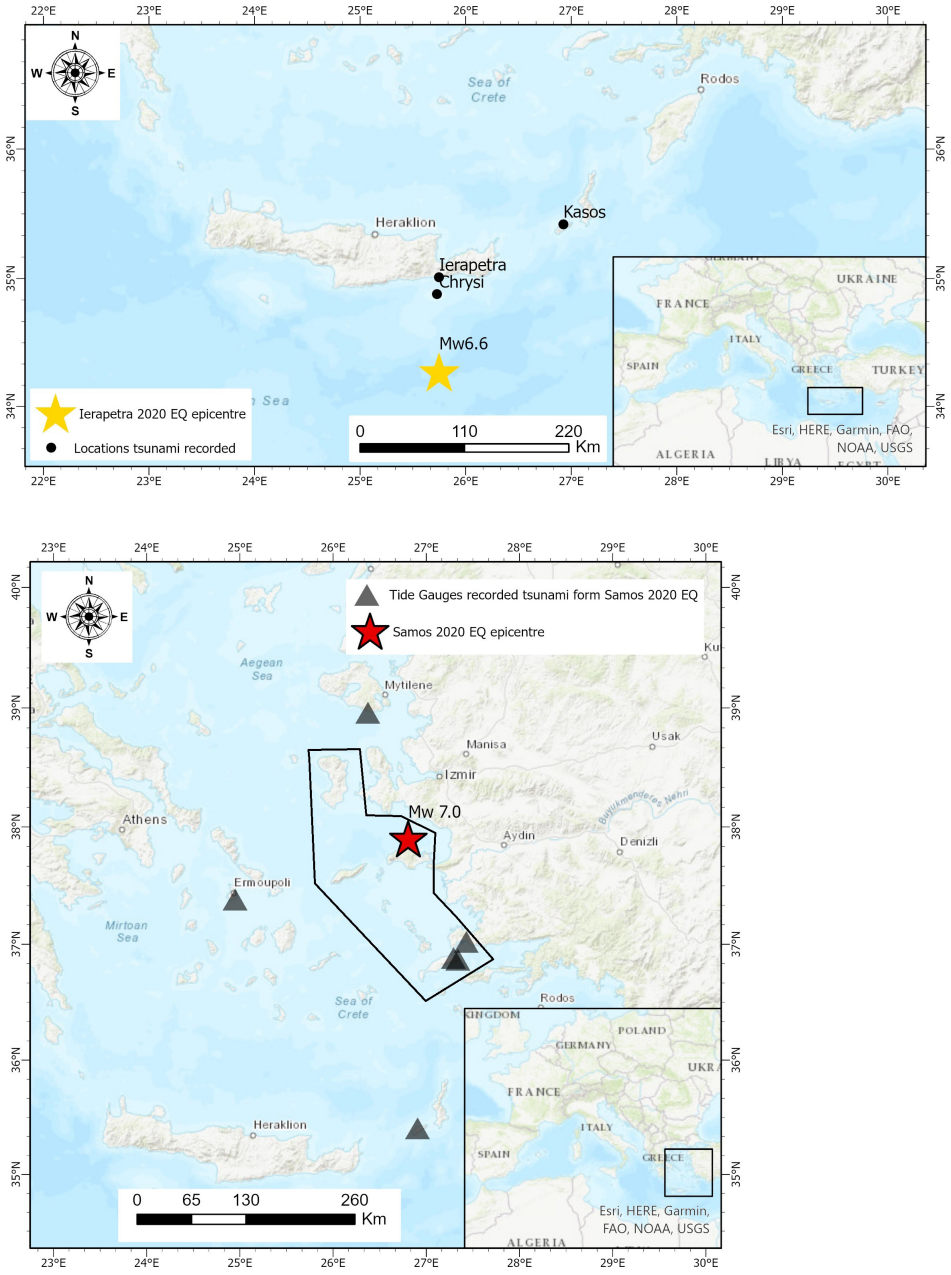
Top: Map of the Ierapetra May 2020 earthquake depicting the epicentre and the locations with tsunami observations. Bottom: The Samos October 2020 earthquake depicting the earthquake epicentre, the tide gauges station that recorded the tsunami (own figure, using data sourced from Triantafyllou *et al*., 2021) and the polygonal area where the 112 emergency warning message was sent. ArcGIS Pro 2 software was used to generate the base maps presented in this figure – all necessary permissions granted.

It was the first time that the emergency number 112 was activated to provide alerts about a potential tsunami in the broader area of Samos island. The warning message about the potential generation of tsunami was issued after the first wave. The emergency number 112 was activated and the first public alerting message was issued at 14:15 local time, approximately 15 minutes after the arrival of the first wave (approximately 25 minutes after the main earthquake), but before the second wave. The message was sent to all cell phones in the islands of Samos, Chios, Ikaria, Fournoi, up to Kos island to the South ( Figure 2). The text was the following: “Stay away from the coast. Danger from high sea waves due to earthquake.”. At 14:45, half an hour later, a second message was sent to the public, informing to stay in open safe places, avoid buildings that suffered damage due to the earthquake and do not overload the phone lines. The fact that the 112-emergency number was activated, even after the first wave, is a step forward in public early warnings.

Interesting is also the response of the general public. From various videos that were published on the web, it is easily inferred that people were not aware of the potential danger to which they were exposed, as they were staring at the sea invading inland instead of moving to higher altitudes. Only during the second wave, which was stronger, and the sea was risen significantly, people started to find safe places to higher altitudes moving away from the coast.

Tsunami travel times are extremely important for the proper warning of the civil protection mechanism and of the population. Tsunami early warning systems are crucial in the planning and notification services. The Southern coasts of Europe are in the Mediterranean Sea, a closed sea in which earthquake epicentres are not far from the coastal zone and thus tsunami travel times are small (example of travel times from Sørensen *et al*., 2012). This is especially the case for Greece.

These two recent events clearly present important gaps related to the instrumentation and real-time simulations, delays in the response and decision making of the civil protection, lack of risk awareness from the general public but also from practitioners, lack in training both for general public and practitioners and lack in standardized ways of elaborating results and warnings especially in a cross-border aspect.

## Identification of future common capability challenges for TWG-natural hazard mitigation

The previously mentioned knowledge and facts on tsunami hazard in Europe and on an international level were employed during the 3rd cycle workshop which was planned in order to identify the future challenges.

### Workshop structure and scenario brief description

The workshop was organized by the TWG-D leader and took place as an online event on the 25 ^th^ of November 2020, putting the gaps of and needs for tsunami risk mitigation into a broader scientific context.

The structure of the workshop was the following:

- Introduction of the FIRE-IN project.
- Introduction of the context of the “Natural Hazards Mitigation” thematic working group of FIRE-IN.
- Overview of the tectonic regime, seismic and tsunamigenic potential in the Mediterranean Sea with a focus on the Eastern Mediterranean Sea and findings from the recent tsunami in Samos.
- Existing warning and alert systems for tsunamis worldwide and most specifically in the Eastern Mediterranean with operational key features.
- Preparedness and the need for education and training of students both in earthquakes and tsunamis.
- Tsunami hazard and risk mapping as a tool for mitigation, especially for critical infrastructure.
- Questions and discussion on the key features of the presented material.

In the workshop, experts and interested stakeholders (first responders, civil protection authorities, representatives of local and regional administration, international organisations and researchers) participated with the aim to discuss gaps and needs.

The scenario was designed to describe the mega earthquake of 365 AD, with a magnitude of Mw=8.3, at the South East of Crete island-Greece in the South Aegean Sea ( Papadimitriou & Karakostas, 2008), which consequently triggered the generation of a tsunami wave. Due to the fact that the workshop took place only one month after the recent Mw=7.0 earthquake in Samos, inevitably a strong focus was given on the discussion of the recent event. The experts briefly presented the current situation regarding the tsunami hazard, installed tsunami warning systems along with global initiatives, education of the public related to tsunamis and land-use planning based on tsunami risk assessment approaches with international examples. The discussion of the recent event, provided to the initial idea a more realistic perspective. Issues regarding the preparedness, response, and recovery phases were also taken into account.

### Workshop focus points per disaster phase

**Figure 3.**
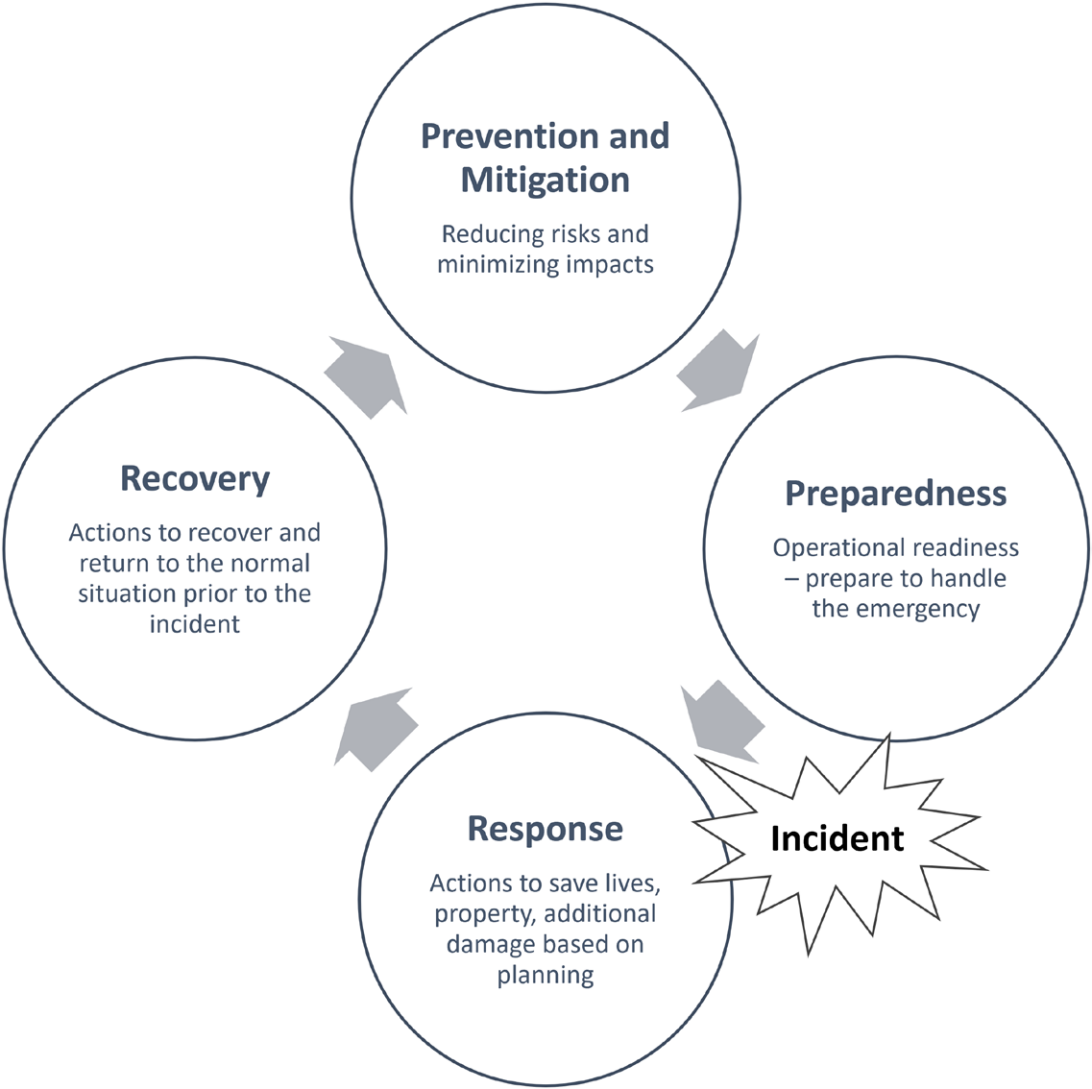
The disaster management cycle.

The scenario was based on the disaster management cycle ( Figure 3) and the results were clustered according to the four phases.

In the prevention and mitigation phase an early warning system is an infrastructure that can provide to people the necessary alerts in order to apply a horizontal or vertical evacuation, moving to higher grounds or gathering at higher floors of buildings. Installation of such systems must be accompanied by the necessary training of both first responders and the population, in order for them to develop the proper behaviour and take advantage of the time frame that an early warning system provides, even if it is a short one.

Regarding the preparedness phase, there are different levels of preparedness if one takes a look around the Mediterranean Sea. There is a gap between the southern Mediterranean countries and North Africa in terms of available infrastructure for earthquake and tsunami monitoring (availability of instruments seismometers, tide gauges and prospects of new algorithms for rapid and accurate determination of earthquake magnitude) as North Africa countries are less prepared, an issue that indirectly affects the Mediterranean countries that belong to the EU as well.

Earthquakes are the type of natural hazard which the time span of an early warning varies from a few seconds to a few minutes depending on the epicentral distance and the earthquake magnitude. Nevertheless, these few seconds maybe enough to save lives. Tsunamis, have larger time span that varies from a few minutes to a few hours depending on the epicentral distance to the coast and the magnitude of the earthquake (considering the fact that the majority of tsunamis is generated by earthquakes). In Mediterranean Sea, coastal densely inhabited areas are prone to tsunamis and the average travel time for a tsunami can be less 15 minutes according to various scenarios provided by Tinti *et al.* (2005). This short time span may not allow the separation of preparedness and response phase. In addition, in some cases, there are high uncertainties in the communication of the tsunami risk, e.g., the earthquake magnitude assessment, which may affect the timely alert of the public, and consequently the proper and fast communication of the imminent risk to the public.

Response is the main phase during which the civil protection mechanism responds in order to save lives. New technologies can assist in these activities, but the responders must be able to act without the help of technology, using only basic tools. Also, help must be distributed to multiple sites usually in complex environment without easy access and at the same time evacuating population and trying to keep alive the ones that stay intact.

In the recovery phase, international experience, lessons learnt from past cases, should be addressed in order for the population to rapidly return to their usual daily lives. Additionally, long-term problems of tsunami impacts, e.g., psychological aspects and resettlement of the population in hazardous areas are some of the problems that arise, require the attention of the state and may extend the time of recovery.

### Challenges related to natural hazards mitigation for the 3rd cycle

Gaps related to the mitigation and preparedness phases were revealed (alerting and warning capabilities of existing systems, vulnerability aspects against tsunamis, education of the public), in the response phase (multiple sites to respond in a complex environment, evacuation of the population) as well as gaps related to the impacts of both earthquake and tsunami, cross-border communication and assistance. The discussion focused on forecasting and simulation of tsunamis and warning systems, simulation exercises, evacuation, the vulnerability of the public, the local emergency managers response and training issues of both public and first responders. In a nutshell, preparedness and proper training are key elements on building resilient societies against severe risks.

Overall, the most important challenges are the following:

1. Anticipate vulnerability and communicate to the public: This is a challenge valid for the future as well, already identified through the 1st cycle of the project ( Lahaye *et al.*, 2018). It is of great importance to develop credible communication channels with the public, as well as to anticipate the potential evolution of emergencies and prioritise actions, synchronising among the different engaged actors and providing a shared understanding. Furthermore, the identification of factors, which could potentially lead to cascading effects and to an unanticipated evolution of the emergency, is crucial. Citizens have to be trained and involved in exercises and drills beforehand in order to know how to react, not to have unrealistic expectations from first responders and to be ready to cooperate. Moreover, when a disaster occurs, they have to be timely and continuously alerted and informed about the ongoing situation and possible sudden changes which may pose threats to the community. In addition, society has to be involved in the negotiations regarding the level of acceptance of risks which are posed by response actions and operations. “Anticipate vulnerability and communicate it to the public” is included in the 3rd cycle challenges “Anticipate and prioritize avoiding the collapse of the emergency system”, “Develop public self-protection and awareness” and the “Negotiate the values with communities before the emergency”.

2. Focus on capacity building towards more resilient societies: This is a challenge that exists and has been identified even from the 1st cycle of the project. But, according to the experts’ panel this is a challenge that will remain for the future as well. Resilient societies are built upon prevention and preparedness. Only, with the proper infrastructure and continuous training, response is more efficient and recovery easier. Past cases must be handled as lessons and areas for improvement. Training involves not only first responders but also the engagement of both scientists and the public in exercises. In addition, training procedures should encompass exercises and drills between first responders who have the know-how of handling specific emergencies and less experienced stakeholders, aiming at the expansion of knowledge and the exchange of experience. The outcomes of scientific research have to be absorbed by the emergency response mechanism and timely communicated not only to relevant stakeholders but also to the public. Doctrines must be developed; best practices must be adopted.

3. Forecast and simulate complex scenarios: science and technology with the use of tools, algorithms, software, proper installed instrumentation and numerous data are needed to be employed so as to forecast imminent risks and run simulations that resemble the reality in order to be more prepared. Miralles *et al*. (2021) examine this challenge under three different angles. First responders have to develop pre-plans that can enable operational flexibility in dynamic emergency situations without limiting the operational window. Moreover, trust, not only among end users, but also between first responders and the public, is an essential ingredient of disaster management. Transparency in decision making is crucial as well as the capability to have a confidant person or group of persons to deliver and transmit important messages. Finally, technology can significantly enhance effective risk management. Technologies span from early warning systems for real or near-real time alert of the community to electronic triage systems and AI tools for the simulation of the evolution of a disaster. Early warning systems are of utmost importance for tsunami emergencies, especially taking into consideration that the timeframe between the tsunami-causing event and the actual arrival of the wave might be extremely narrow. “Forecast and simulate complex scenarios” is a CCC falling under the umbrella of the Technology Capability of the 1st cycle. This challenge presents strong links with the FCCC “Focus on governance and integral risk management” of the Preparedness Capability.

## 3rd cycle overall results and discussion

During the third cycle of workshops, for the identification of common and future common capability challenges, modifications to the challenges and capabilities were implemented. The most important modification was that, although the four (4) main Challenges remained the same as in the previous cycles i.e., High Flow of resources in a hostile environment scenario, High Impact Low Frequency (HILOF) scenarios, Multiagency / Multileadership (MA/ML) scenarios and high level of Uncertainty (UN), some of the Capabilities were rearranged and renamed in order to better depict the results of the conversations that took place among the experts during the third cycle workshops. According to Miralles *et al*. (2021) the Capabilities of the 3rd cycle are the following six:

- Incident Command Organization
- Preparedness
- Risk Reduction
- Knowledge Cycle
- Community Involvement
- Decision Making Cycle

Overall, 24 Capability Challenges were identified. Among them, 14 are considered as current challenges that were identified already from the previous cycles, but still remain largely unresolved. The other 10 challenges have a more prospective character and are expected to concern first responders both in the short and the long term, especially taking into consideration the rapid and continuous increase in the frequency and severity of disasters. The majority of FCCCs are associated with the main challenge of Uncertainty, something that comes as no surprise, since large scale emergencies are often accompanied by sudden changes, which can disrupt operations and cause confusion between the several different first responders’ agencies. Overall, the CCCs of the 1st cycle and the prioritised CCCs of the 2nd cycle are by no means negated, but they are rather absorbed and incorporated into the challenges of the 3rd cycle. Table 1 depicts the 3rd cycle and final matrix of Common and Future Common Capability Challenges.

**Table 1.** The 3 ^rd^ cycle Common and Future Common Capability Challenges. CCCs (Common Capability Challenges) are coloured in black while FCCCs (Future Common Capability Challenges) are coloured in blue. ( Miralles *et al*., 2021).

TABLE 1	High flow of effort in hostile environment (HF)	High Impact, Low Frequency (HILOF)	Multi-agency / Multi- leadership (ML)	High level of uncertainty (UN)
**Incident** **Command** **Organization**	CCC-1. Organize to sustain safe operations.	CCC-2. Anticipate and prioritize avoiding the collapse of the emergency system.	CCC-3. Build interoperability for a distributed decision- making based on a shared understanding of the emergency.	FCCC-4. Strategic management focused on proactively reducing sources of uncertainty and building robustness and resiliency.
**Community** **involvement**	CCC-5. Develop public self- protection and awareness.	CCC-6. Involve communities and key stakeholders as active actors in risk management.	FCCC-7. Negotiate the values with communities before the emergency.	FCCC-8. Cultural change towards risk tolerance and resilience.
**Knowledge** **Cycle**	CCC-9. Train specific roles and risks and invest in a robust knowledge cycle	FCCC-10. FRS empowered to innovate and build organizational learning	CCC-11. Build a shared understanding of the emergency, and train interagency scenarios	FCCC-12. Focus on capacity building towards more resilient societies
**Decision** **Making Cycle**	CCC-13. Make operational decisions based on building an understanding of the emergency and its evolution	CCC-14. Choose a strategical scenario of resolution, and distribute tactical decision-making	CCC-15. Build a shared understanding of the scenario to synchronize decision-making	FCCC-16. Create certainty and shared vision of emergencies.
**Risk** **reduction**	CCC-17. Focus encouraging self-capacities and safety	CCC-18. Negotiate solutions with stake holders for anticipated scenarios	FCCC-19. Integrate risk prevention and safety into other policies and actors	FCCC-20. Focus on governance and integral risk management.
**Preparedness**	CCC-21. Pre-plan a time- efficient, safe response, minimizing responder’s engagement	CCC-22. Plan in a more integral way	FCCC-23. Pre-plan interoperability and enhance synergies	FCCC-24. Focus on governance and integral risk management.

FIRE-IN based on the challenges and gaps, makes one step further and identifies potential solutions for the identified challenges and proposes how well covered is the challenge according to the traffic light system on the three pillars of technology, research items and existing standards. This is a process of interaction between practitioners, researchers and industry in order to bring them all together and find the level of coverage of challenges and consequently provide information for future research programming.

The interaction between practitioners, researchers and industry was enhanced through online questionnaires and an online workshop that took place on January 2022 in the framework of the FIRE-IN project ( Tsaloukidis *et al.*, 2022), in order to boost the discussion of the current and future challenges and seek for future ideas that could solve the problems of practitioners in the daily operations. These questionnaires were addressed to technological providers and first responders and examined the importance of technology and standardisation in disaster management, technological solutions and their necessity for first responders’ operations and the level of significance of the 3rd cycle CCCs and FCCCs from the providers’ point of view. Table 2 depicts the level of coverage of the 3rd cycle challenges by technological innovations (T), research projects and papers (R) and standardisation activities (S), as the outcome of the solution screening and the request for ideas processes, both conducted under the framework of the project.

**Table 2.** Level of coverage of CCCs (Common Capability Challenges) and FCCCs (Future Common Capability Challenges). T refers to technological solutions, R to research items and S to standardisation items. Green colour corresponds to a challenge well covered in the corresponding sector with mature solutions, yellow denotes that solutions exist but are in a state of development and not mature enough, while red denotes that the topic is being addressed but the solutions are limited and the challenge is not covered ( Tsaloukidis *et al*., 2022).

TABLE 2	High flow of effort in hostile environment (HF)	High Impact, Low Frequency (HILOF)	Multiagency / Multileadership (ML)	High level of uncertainty (UN)
**Incident** **Command** **Organization**	CCC-1. Organize to sustain safe operations	CCC-2. Anticipate and prioritize avoiding the collapse of the emergency system	CCC-3. Build interoperability for a distributed decision- making based on a shared understanding of the emergency	FCCC-4. Strategic management focused on proactively reducing sources of uncertainty and building robustness and resiliency.
	T	T	T	T
	R	R	R	R
	S	S	S	S
**Community** **involvement**	CCC-5. Develop public self- protection and awareness	CCC-6. Involve communities and key stakeholders as active actors in risk management	FCCC-7. Negotiate the values with communities before the emergency	FCCC-8. Cultural change towards risk tolerance and resilience.
	T	T	T	T
	R	R	R	R
	S	S	S	S
**Knowledge Cycle**	CCC-9. Train specific roles and risks and invest in a robust knowledge cycle	FCCC-10. FRS empowered to innovate and build organizational learning	CCC-11. Build a shared understanding of the emergency, and train interagency scenarios	FCCC-12. Focus on capacity building towards more resilient societies
	T	T	T	T
	R	R	R	R
	S	S	S	S
**Decision Making** **Cycle**	CCC-13. Make operational decisions based on building an understanding of the emergency and its evolution	CCC-14. Choose a strategical scenario of resolution, and distribute tactical decision-making	CCC-15. Build a shared understanding of the scenario to synchronize decision-making	FCCC-16. Create certainty and shared vision of emergencies.
	T	T	T	T
	R	R	R	R
	S	S	S	S
**Risk reduction**	CCC-17. Focus encouraging self-capacities and safety	CCC-18. Negotiate solutions with stake holders for anticipated scenarios	FCCC-19. Integrate risk prevention and safety into other policies and actors	FCCC-20. Focus on governance and integral risk management.
	T	T	T	T
	R	R	R	R
	S	S	S	S
**Preparedness**	CCC-21. Pre-plan a time- efficient, safe response, minimizing responder’s engagement	CCC-22. Plan in a more integral way	FCCC-23. Pre-plan interoperability and enhance synergies	FCCC-24. Focus on governance and integral risk management.
	T	T	T	T
	R	R	R	R
	S	S	S	S

According to Tsaloukidis *et al*. (2022), who analyses the results of the “Request for Ideas” procedure and the results of the workshop, the future challenges “Anticipate and prioritize avoiding the collapse of the emergency system”, “Develop public self-protection and awareness” and “Negotiate the values with communities before the emergency”, which are closely related to the 1st cycle challenge “Anticipate vulnerability and communicate to the public”, are addressed by a significant number of solutions. Technological solutions and also research do, in fact, examine interactions and communication with the public. The interpretation of a variety of solutions addressing these challenges could be twofold, firstly that indeed the public plays an important role in all phases of the disaster management cycle, and this is something acknowledged by researchers and technological providers, and, secondly, that all of these challenges are well addressed and well covered by solutions.

Consequently, the uptake and adoption of technological solutions by first responder organizations needs to be encouraged. Additionally, standards, dealing with the issue of interaction with the public, do exist.

On the other hand, “Focus on capacity building towards more resilient societies” is a challenge that seems to require more intensive research, as the solutions addressing it are significantly low. However, this challenge, having a more theoretical character, as it is related to strategies for the enhancement of the capacity of the community, requires a different, more institutional approach, and the fact that it cannot be addressed by technological innovations comes as no surprise. The same also applies to the “Focus on governance and integral risk management” of the Risk Reduction Capability. Tsaloukidis *et al*. (2022) state that research and standardisation can help to identify and develop solutions which could pave the way for novel policies and doctrines, capable to strengthen disaster risk management. On the other hand, “Focus on governance and integral risk management” of the Preparedness capability is closely linked to the development of cutting-edge technologies and, as a matter of fact, a large number of technological solutions addressing this challenge is screened in the context of the project.

Early warning technologies, inter alia, possess a prominent position among technological providers and first responders and are considered quite essential, both in the mid- and the long-term, for the timely alert of citizens and, therefore, for efficient response operations ( Tsaloukidis *et al*., 2022).

On the other hand, the capability challenges regarded as crucial for the tsunami scenario seem to be of a lesser concern for technological providers. This conclusion is without surprise, considering that the aforementioned challenges focus on a change of perspective from the population and not on the technological angle. The involvement of the public in disaster management processes for the enhancement of its awareness and capacity, and especially in actions related to pre-disaster phases i.e., mitigation-prevention and preparedness, has to be addressed at the top level and this is not primarily a matter of technology but rather of political will and strategical planning.

The involvement of the community and its identified challenges was highly discussed as there seems to be a strong agreement between practitioners and researchers, regarding the participation of the public in disaster management procedures and the need for citizens to consider themselves as active players and not passively await their rescue in case of emergencies. Additionally, “Anticipate and prioritize avoiding the collapse of the emergency system”, a challenge highlighted during the tsunami workshop, has been also recognized of importance by providers and end users who took part in the online workshop.

## Recommendations and next steps

Following the definition of Decision 1313/2013/EU, “preparedness is the state of readiness and capability of human and material means, structures, communities and organisations, enabling them to ensure an effective rapid response to a disaster”, preparedness measures are elements of emergency management, but they can also be structural or non-structural measures implemented for reducing risk, such as early warning systems or training procedures. Following the same Decision and its amendment (2019/420/EU), each Member State should submit to the EC every three years a national risk assessment and reports on available risk management capabilities and planning, in which special reference is made to the low probability risks with a high impact and more particularly the implemented and planned priority prevention and preparedness measures.

As described in the previous chapters of this article, the most crucial issues to be addressed in the near-future that have been emerged, especially for the case of tsunamis, are the following: (a) the complete coverage of the seas surrounding Europe by sensors for the timely notification of operational centers, along with advancements in public early warning systems, with the aim the alerting message to reach exposed populations as soon as possible, and (b) the change of perspective of the population towards preparedness and resilience in order to be treated as active members of the disaster management procedures. While for the first it is a matter of science, research and technological advancements, for the latter it is also a matter of political will and cultural change. It is of great importance to promote policies, which will focus on the training of both the general population and first responders’ organizations in the face of complex and dynamic situations with unexpected evolution, for which there is no relevant experience and competence. This turn to preparedness from the understanding and awareness of the imminent threat and risk to a specific action on a self-protection level to supporting and helping the others and be prepared to expect the unexpected is valid not only for tsunamis but also for other risks. The society as a whole, from pupils to citizens, scientists, experts and first responders must be constantly trained and educated to be always prepared. Focus on preparedness, with all of its components, is the new policy and recommendation.

## Ethics and consent

Ethical approval and consent were not required.

## Data Availability

No data are associated with this article.
