# Supplementary material for: The FIRE-IN project: Tsunami-risk related practitioner challenges and 3rd cycle overall results
Source: Open Res Eur. 2024 May 17;3:5. Originally published 2023 Jan 10. [Version 3] doi: 10.12688/openreseurope.15249.3 (PMC10858983; doi:10.12688/openreseurope.15249.3)
Supplement: Peer review history

# Reviewer report: Reviewer response for version 3

**DOI:** 10.21956/openreseurope.19007.r40646

**Published:** 2024-05-28

**Authors:** Yong-Jun Lin

**Affiliations:** 1 Center for Weather and Climate Disaster Research, National Taiwan University, Taipei, Taiwan

I think this revision addressed my comments.

Where applicable, are recommendations and next steps explained clearly for others to follow? (Please consider whether others in the research community would be able to implement guidelines or recommendations and/or constructively engage in the debate)

Yes

Does the article adequately reference differing views and opinions?

Yes

Are all factual statements correct, and are statements and arguments made adequately supported by citations?

Yes

Is the rationale for the Open Letter provided in sufficient detail? (Please consider whether existing challenges in the field are outlined clearly and whether the purpose of the letter is explained)

Partly

Is the Open Letter written in accessible language? (Please consider whether all subject-specific terms, concepts and abbreviations are explained)

Yes

Reviewer Expertise:

Early Warning Systems, Resilience Community, Risk Map

I confirm that I have read this submission and believe that I have an appropriate level of expertise to confirm that it is of an acceptable scientific standard.

# Reviewer report: Reviewer response for version 2

**DOI:** 10.21956/openreseurope.17752.r36688

**Published:** 2024-02-20

**Authors:** Maria Polese

**Affiliations:** 1 University of Naples Federico II, Naples, Italy

Within the FIRE-IN project a methodology for identification of practitioners challenges was developed. This paper presents the results of application of the methodology with a particular focus on natural hazard mitigation issues. The scenario of occurrence of a tsunami in the Mediterranean sea was adopted as a basis for a case study to be discussed in a workshop involving experts and interested stakeholders (e.g. fist responders, civil protection authorities, representatives of local and regional administrations and researchers).

 The methodology is founded on an cyclic process that entails the involvement of stakeholders clustered along relevant Thematic Working Groups (TWG). The so-called Common Capability Challenges (CCC) where identified in the first two cycles of work, while the third cycle, for which the results are presented in the present paper, deals with Future Common Capability Challenges (FCCC).

 The overall results appear to be solid and based on confrontation with a wide network of stakeholders, ensuring the potential interest of the results.

 However, the paper needs to be improved since there are quite some drawbacks that hamper its readability and clearness for the reader.

 Below some detailed comments:

1. The abstract anticipates some wording that may not be clear to the reader. As it is now, the abstract is not very much informative since “working group” and “3 ^rd^ cycle” are not yet defined. It is suggested to review the abstract so to let it be comprehensible without knowing the project and the methodology that will be explained in the next sections. The abstract should be self-consistent
2. Throughout the text there are some references to deliverables or project outputs that do not add information and are not necessary, at least as they are referred now. For example, at the end of the Introduction, the following sentence is included “A scenario of mega-earthquake in the Mediterranean with a cascading tsunami that can affect a significant part of Europe is the basis for the discussion, review and elaboration of the results of the 3rd cycle of workshops for the Natural Hazard Mitigation TWG (D) as already defined in respective project deliverables (Miralles et al., 2021).” It would be better to simply reformulate: “A scenario of mega-earthquake in the Mediterranean with a cascading tsunami that can affect a significant part of Europe is the basis for the discussion, review and elaboration of the results of the 3rd cycle of workshops for the Natural Hazard Mitigation TWG (D); more details may be found in (Miralles et al., 2021).”
3. Reference is made to ACRIMAS taxonomy and framework without giving any explanation. Some lines explaining what is the taxonomy about and also on the framework should be added, otherwise the reader cannot understand what is taken from this project.
4. Authors should be careful to be consistent in the terminology.
  - For example, at the end of the Introduction, reference is made to “new” capability challenges for practitioners. Is it intended as Future Capability challenges, consistently to FCCC introduced before?
  - Also, when the General Framework is presented, at the beginning of the paragraph the authors refer to preparatory, operational and common tasks….however, few lines below reference is made to operational, supporting and preparatory tasks….are the concepts the same? If so the same wording should be adopted to avoid confusion
5. At the end of description of the general framework there is the following sentence: “Operational and supporting tasks refer to the response and recovery phases, while preparatory tasks to mitigation and preparedness.” It would be nice to try to connect the tasks with the phases of the Disaster Risk Management DRM cycle in a Figure, similar to Figure 4. In principle, the very same figure could be used to show both concepts, the phases of DRM and the type of tasks. Hence, it is suggested to introduce already at this stage of description of the framework the important distinction of the different phases of DRM and to propose the connection with the tasks ina Figure. Then, Fig. 4 would not anymore needed where it is now.
6. The final part of the sentence that ends with “…the opportunities from various perspectives such as, procedures, personnel, equipment, tools and technologies” is too vague. What does this mean? Please specify better.
7. At the end of the paragraph Identification of CCCs through workshops it is mentioned that specific scenarios are used as a base for discussion. Authors should elaborate more on how such specific scenarios are built, which criteria are used for their development etc.
8. The “research”, “standardisation” and “technology” domains are introduced without a real definition or explanation. Authors should give a definition and provide few preliminary examples for the reader to better comprehend.
9. In explaining the traffic light system authors argue that the challenges are examined considering “certain criteria”. Which are such criteria? Please elaborate more on such concept.
10. Please add reference for CEN/CENELEC
11. It is suggested to better organize the numbering of chapters/subchapters e.g. introducing numbers or different style for titles and sub-titles. For example when referring to Recent operational experience from Greece, the following paragraph on Ierapetra earthquake and Samos earthquake should be clearly recognizable as sub-paragraphs but actually the font title is the same as the main paragraph.
12. Please explain what is intended as horizontal and vertical evacuation
13. In the paragraph Workshop focus points per disaster phase, authors comment that there is a gap between southern mediterranean countries and North Africa…however, such statement is not supported by any example on the preparedness phase, while there are comments on early warning, that according to the authors refer to the prevention and mitigation phase.
14. In the sentence “FIRE-IN based on the challenges and gaps, makes one step further and identifies potential solutions for the identified challenges and proposes how well covered is the challenge according to the traffic light system on the three pillars of technology, research items and existing standards (see Table 3)” Correct reference should be to Table 4 (not Table 3)
15. The recommendation and next steps are interesting; however, it seems mostly that they are a result of the studies performed in preparation of the workshop and partly on  results of the workshop. On the other hand, the authors should make an effort to connect such observations to the  FCCC and solutions, that at the moment are not recalled and do not appear in such conclusive paragraph.
16. In general the English language should be reviewed, since there are several parts of the paper that are not clear or where the sentences are not grammatically correct. For example the sentence “The “Request for Ideas” is a process in which relevant to crisis to research and development stakeholders can provide information and solutions that can address specific CCCs or FCCCs, either in the form of an existing solution or in the form of a future product” should be rewritten

Where applicable, are recommendations and next steps explained clearly for others to follow? (Please consider whether others in the research community would be able to implement guidelines or recommendations and/or constructively engage in the debate)

Partly

Does the article adequately reference differing views and opinions?

Yes

Are all factual statements correct, and are statements and arguments made adequately supported by citations?

Yes

Is the rationale for the Open Letter provided in sufficient detail? (Please consider whether existing challenges in the field are outlined clearly and whether the purpose of the letter is explained)

Partly

Is the Open Letter written in accessible language? (Please consider whether all subject-specific terms, concepts and abbreviations are explained)

Partly

Reviewer Expertise:

Seismic vulnerability, risk/loss estimation methodologies, multi-risk assessment, crisis management policies framework

I confirm that I have read this submission and believe that I have an appropriate level of expertise to confirm that it is of an acceptable scientific standard, however I have significant reservations, as outlined above.

# Response

**Published:** 2024-03-30

**Authors:** Georgios Sakkas

The authors would like to thank the reviewer (4) for the constructive comments that help to make the concept of the FIRE-IN project, the work carried out and the results and outcomes clearer to a broader audience. Answers: Abstract has been improved. Second comment addressed. Reformulaiton has been made. Please the document with the track changes. The new description provided more details on the ACRIMAS methodology being at the same time concise enough. The word “new” was added by mistake, proper explanations are given. This is a similar comment from reviewer 2. Named common by mistake. Corrected to “supporting”. This is a similar comment to reviewer 2. Figure 4 was moved in the beginning and made Figure 1. Tasks were added in the disaster management cycle. Changed to the following “…the various opportunities for improvements in procedures, personnel, new equipment, tools and technologies.” This paragraph is now ending with the following statement “This procedure is based on a deterministic scenario which arises from past events or from hypothetical future events or even a worst-case scenario.”. In addition, for the 3rd cycle a detailed justification on the selection of the respective scenarios is already given in the chapter “TWG-Natural Hazards Mitigation: Tsunamis as a case study for Europe”. In the same paragraph, examples of the research, technology and standardisation domains are given. Other than that we believe that the terms are self-explanatory. CEN and CENELEC are two of the three European Standardisation Organizations officially recognized by the EU and by teh European Free Trade Associations as being responsible to develop standards at European level. It is an organization that does not need reference. To make it easier we added a direct link to the official website. Only the “Ierapetra earthquake” and “Samos earthquake” had different heading probably an error in the different versions of the document. Now this is corrected, and the two sub-chapter shave the correct heading level. About the evacuation (horizontal & vertical): Although we find it as self-explanatory, the comment is properly addressed in the respective section. Correct reference to table 4 made. We specifically wanted to present the results of the project. Nevertheless, improvements have been made to the last sections of the diocument. In general the whole document was revised to identify potential grammatical and syntax errors.

# Reviewer report: Reviewer response for version 2

**DOI:** 10.21956/openreseurope.17752.r34336

**Published:** 2024-02-10

**Authors:** Jörn Lauterjung

**Affiliations:** 1 Deutsches GeoForschungsZentrum GFZ, Potsdam, Germany

I have no further comments for this paper

Where applicable, are recommendations and next steps explained clearly for others to follow? (Please consider whether others in the research community would be able to implement guidelines or recommendations and/or constructively engage in the debate)

Partly

Does the article adequately reference differing views and opinions?

Yes

Are all factual statements correct, and are statements and arguments made adequately supported by citations?

Yes

Is the rationale for the Open Letter provided in sufficient detail? (Please consider whether existing challenges in the field are outlined clearly and whether the purpose of the letter is explained)

Yes

Is the Open Letter written in accessible language? (Please consider whether all subject-specific terms, concepts and abbreviations are explained)

Yes

Reviewer Expertise:

Geosciences, Early Warning Systems

I confirm that I have read this submission and believe that I have an appropriate level of expertise to confirm that it is of an acceptable scientific standard.

# Reviewer report: Reviewer response for version 2

**DOI:** 10.21956/openreseurope.17752.r36696

**Published:** 2024-01-08

**Authors:** Annisa Triyanti

**Affiliations:** 1 Utrecht University, Utrecht, Netherlands Antilles

The manuscript has highlighted an important and timely topic on tsunami risk. It has a high potential value to fill in the knowledge gap on tsunamis as low probability-high impact risks with a cascading and uncertain nature. The manuscript presents the landscape of tsunami risk in Europe with different triggers, current capacity, and challenges in the future, especially for practitioners. I commend the authors for their completeness in presenting historical and geographical data and learning from projects of a similar nature, not only in European but also global south contexts (e.g., the case of Indonesia). I enjoyed reading the manuscript as it has a very clear and coherent storyline and uses accessible language.

 With that, I have two fundamental comments to be addressed by the authors: first is regarding the general framework, and second is regarding the presentation of scientific data/literature.

 1. The general framework is not fully elaborated under the methodology section, including the definition of each sub-element and also how precisely this general framework links to the identification of CCCs and screening and request for ideas. My suspicion is that this has been discussed in detail in the other project deliverable (?). However, I believe that it is important to elaborate more, especially since the methodology is the key purpose of the article, and the general framework is used to explain the results. Furthermore, it is not clear why this particular framework is used, why it fits this research, why the authors did not combine it with other concepts/frameworks that perhaps would offer more diverse perspectives.

 2. There is space to cite more literature, especially when discussing the governance aspect of tsunami risks mentioned as key responses (under the section 3rd cycle overall results and discussion): e.g., public awareness, risk communication, capacity building. It would be good if this article not only refers to its results but also discusses how the results/findings of this project contribute to the existing body of knowledge on this topic.

 Finally, as a clarification point, please indicate to which actors to target the recommendations, if practitioners, which ones and at which level? The article has only vaguely mentioned the actors (practitioners, researchers, industries, member states).

Where applicable, are recommendations and next steps explained clearly for others to follow? (Please consider whether others in the research community would be able to implement guidelines or recommendations and/or constructively engage in the debate)

Yes

Does the article adequately reference differing views and opinions?

Partly

Are all factual statements correct, and are statements and arguments made adequately supported by citations?

Partly

Is the rationale for the Open Letter provided in sufficient detail? (Please consider whether existing challenges in the field are outlined clearly and whether the purpose of the letter is explained)

Yes

Is the Open Letter written in accessible language? (Please consider whether all subject-specific terms, concepts and abbreviations are explained)

Yes

Reviewer Expertise:

disaster risk, climate risk, governance, co-production of knowledge, global south

I confirm that I have read this submission and believe that I have an appropriate level of expertise to confirm that it is of an acceptable scientific standard, however I have significant reservations, as outlined above.

# Response

**Published:** 2024-03-30

**Authors:** Georgios Sakkas

The authors would like to thank the third reviewer for the constructive comments that help to make the concept of the FIRE-IN project, the work carried out and the results and outcomes clearer to a broader audience.

- The description of the general framework has been improved, giving more details. Also, the sequence of the sub-chapters changed as we moved the “Common Capability Challenges” sub-chapter exactly after the “Identification of CCCs through workshops”.
- Now, the text is easier to read and mirrors better the rationale behind it.
- Some literature was added in the respective section. Improvements have been made based on the reviewer comment.
- About the target groups: This is added in the recommendations section. Also, it is mentioned in the last rows of the introduction section.

# Reviewer report: Reviewer response for version 2

**DOI:** 10.21956/openreseurope.17752.r36692

**Published:** 2024-01-02

**Authors:** Yong-Jun Lin

**Affiliations:** 1 Center for Weather and Climate Disaster Research, National Taiwan University, Taipei, Taiwan

The draft- "The FIRE-IN project: Tsunami-risk related practitioner challenges and 3rd cycle overall results" focuses on the FIRE-IN (Fire and Rescue Innovation Network) project, funded under the Horizon 2020 program. The project aims to improve European fire and rescue capabilities by establishing a network of practitioners, technology providers, and researchers. It emphasizes identifying and addressing challenges in natural hazard mitigation, mainly focusing on tsunami risks in the Mediterranean region. The document outlines the methodology for identifying challenges, the scenario of a potential Mediterranean tsunami, and the importance of preparing for high-impact, low-probability events. It discusses current European and international measures for tsunami risk mitigation and the need for enhanced preparedness and resilience. The project's outcomes are intended to guide European tsunami-related disaster management stakeholders towards greater safety and resilience in the future.

 The open letter is well written. Minor comments are listed below:

1. In “the general framework”, the authors stated that “management functions are clustered in “preparatory”, “operational” and “common” tasks” in the first paragraph. However, in the following paragraph, operational and preparatory tasks are described and supporting ones. How about the common tasks? Or supporting tasks are common tasks?
2. What does “TRL” refer to in Table 1?
3. Figure 2 shows the selected 13 tsunami sources corresponding to those of Table 2 in red numbers. Making the number in the star is recommended to make a difference from the colored rectangles with numbers. Moreover, the star is too large to make some rectangles hard to see. Furthermore, what are those rectangles? In the legend, they referred to tsunamigenic zones; the description is in the figure captions. Please describe in the texts for easy reading.
4. Figure 4 is great. Do you have any citation of this figure, e.g. FEMA? However, it may be introduced in the front of the open letter. The tasks mentioned in Comment 1 are described in their corresponding phases.

Where applicable, are recommendations and next steps explained clearly for others to follow? (Please consider whether others in the research community would be able to implement guidelines or recommendations and/or constructively engage in the debate)

Yes

Does the article adequately reference differing views and opinions?

Yes

Are all factual statements correct, and are statements and arguments made adequately supported by citations?

Yes

Is the rationale for the Open Letter provided in sufficient detail? (Please consider whether existing challenges in the field are outlined clearly and whether the purpose of the letter is explained)

Partly

Is the Open Letter written in accessible language? (Please consider whether all subject-specific terms, concepts and abbreviations are explained)

Yes

Reviewer Expertise:

Early Warning Systems, Resilience Community, Risk Map

I confirm that I have read this submission and believe that I have an appropriate level of expertise to confirm that it is of an acceptable scientific standard, however I have significant reservations, as outlined above.

# Response

**Published:** 2024-03-30

**Authors:** Georgios Sakkas

The authors would like to thank the second reviewer for the constructivw comments that helped to make the concept of the FIRE-IN project, the work carried out and the results and outcomes clearer to a broader audience. Answer:

- Common tasks are the supporting tasks and it was a mistake of the main author. In this version it is corrected.
- TRL explanation has been added in the caption of Table 1.
- Figure 2 has been improved. It was also added in the text. In addition, Figure 2 is now Figure 3 due to a change in the figures sequence, see also answer to the next comment.
- Figure 4 was moved in the beginning, and now in version 3 of the document, it is figure 1, following a similar comment of reviewer 4.

# Reviewer report: Reviewer response for version 1

**DOI:** 10.21956/openreseurope.16491.r30602

**Published:** 2023-01-26

**Authors:** Jörn Lauterjung

**Affiliations:** 1 Deutsches GeoForschungsZentrum GFZ, Potsdam, Germany

The article summarizes results gained during the project FIRE-IN, a Horizon 2020 project that establishes and supports a network of practitioners, technological providers and researchers with a main goal to improve the national and European Fire and Rescue capability development process and consequently enhance the security level of the citizens of the European Union. Through its outcomes, the project aims at supporting the European Commission policy makers to build a Research and Innovation Agenda. Main activities within the project are (a) the identification and harmonization of operational capability gaps, (b) the scouting of promising solutions, (c) the request for ideas by the community and the various relevant stakeholders and (d) the definition of a Fire and Rescue Strategic Research and Standardization Agenda.

 Tsunamis as a risk for Europe are taken as a case study for Europe during the 3rd cycle of the project. Mitigation and preparedness actions in Europe and worldwide related to tsunamis as well as the recent operational experience due to earthquake induced tsunamis in Greece and the Aegean Sea are described. Tsunamis are examined as a major potential threat (high impact – low probability events) for Europe for the next 10 to 15 years with significant future capability challenges for practitioners.

 A scenario of mega-earthquake in the Mediterranean with a cascading tsunami that can affect a significant part of Europe is the basis for the discussion, review and elaboration of the results. The identified future common capability challenges are presented and discussed. Recommendations are presented.

 Overall this is a well written article, although some copyediting is recommended. There are some specific comments and recommendations concerning the text.

1. It is recommended to include a map of Europe with all the mentioned historical and recent tsunamis and a table with their main characteristics instead of a sometimes lengthy textual recital.
2. On page 9, the activation of a phone number 112 is described. Please help the reader to understand the idea and the concept behind phone number 112. This is not clear by the text.
3. The main outcomes are presented in table 1 and table 2. The contents of table 1 somehow follow from the discussed context but for table 2 some discussion of the colour rows for technical solutions, research items and standardisation items is missing. While the colours for technical and research items are understandable, the green colour for standardization for **each **CCC and FCCC is not clear. What is meant by this? For example there are some cases with red for technological solutions and research item but green for standardization which means there is no technical solution, there is still research to do but the standardization is already known and in place. This seems difficult to understand without some explanation. Maybe it would help to include a definition of what is meant by standardization.
  In addition it is recommended to leave positions white or empty if it cannot be addressed by the respective issue (e.g. technical solutions for FCCC 8 or FCCC 12)   

Where applicable, are recommendations and next steps explained clearly for others to follow? (Please consider whether others in the research community would be able to implement guidelines or recommendations and/or constructively engage in the debate)

Partly

Does the article adequately reference differing views and opinions?

Yes

Are all factual statements correct, and are statements and arguments made adequately supported by citations?

Yes

Is the rationale for the Open Letter provided in sufficient detail? (Please consider whether existing challenges in the field are outlined clearly and whether the purpose of the letter is explained)

Yes

Is the Open Letter written in accessible language? (Please consider whether all subject-specific terms, concepts and abbreviations are explained)

Yes

Reviewer Expertise:

Geosciences, Early Warning Systems

I confirm that I have read this submission and believe that I have an appropriate level of expertise to confirm that it is of an acceptable scientific standard, however I have significant reservations, as outlined above.

# Response

**Published:** 2023-07-27

**Authors:** Georgios Sakkas

The authors would like to thank the reviewer for his constructive comments which made the updated version more clear. 1. In the document Table 2 and figure 2 were added. Figure 2 is a modified map of Papadopoulos et al., 2014 (see refs) with the sources mentioned in Table 2. 2. A description for 112 was added, please check page 17 (middle of page). 3. To explain better and make clear the whole concept, a new Table 1 was added that describes the traffic light system (please see pages 6 and 7). Standardization is explained in pages 7 below Table 1. Moreover, below Table 4 (table 2 in version 1) additional explanation is provided. A final note: We cannot leave a cell without a colour/empty because the traffic light system foresees three specific colours (check Table 1 in the new version).
